# Compressive Creep and Shrinkage of High-Strength Concrete Based on Limestone Coarse Aggregate Applied to High-Rise Buildings

**DOI:** 10.3390/ma14175026

**Published:** 2021-09-02

**Authors:** Euichul Hwang, Gyuyong Kim, Kyungmo Koo, Hyungjae Moon, Gyeongcheol Choe, Dongkyun Suh, Jeongsoo Nam

**Affiliations:** 1 Department of Architectural Engineering, Chungnam National University, 99 Daehak-ro, Daejeon 34134, Korea; sksdmlcjf@naver.com (E.H.); speed1382@gmail.com (G.C.); syhtw@naver.com (D.S.); j.nam@cnu.ac.kr (J.N.); 2 Building Engineering Team, Hanwha Engineering & Construction, 86 Cheonggyecheon-ro, Seoul 04541, Korea; kkm@hanwha.com; 3 R&D Center, Lotte E&C, Seoul 06515, Korea; doorbrother@lotte.net

**Keywords:** high-strength concrete, compressive strength, elastic modulus, autogenous shrinkage, drying shrinkage, compressive creep

## Abstract

Concrete undergoes shrinkage regardless of the influence of external forces. The deformation of concrete is crucial for the structural stability of high-rise and large-scale buildings. In this study, the shrinkage and compressive creep of 70–90 MPa high-strength concrete used in high-rise buildings were evaluated based on the curing conditions (sealed/unsealed), and the existing prediction models were examined. It was observed that the curing condition does not significantly affect the mechanical properties of high-strength concrete, but the use of limestone coarse aggregate increases the elastic modulus when compared to granite coarse aggregate. The autogenous shrinkage of high-strength concrete is greater than that of normal-strength concrete owing to self-desiccation, resulting in a large variation from the value predicted by the model. The drying shrinkage was observed to be similar to that predicted by the model. Compressive creep was affected by the curing conditions, compressive strength, loading level, and loading age. The compressive creep of high-strength concrete varied significantly from the prediction results of ACI 209; ACI 209 was modified based on the measured values. The shrinkage and compressive creep characteristics of high-strength concrete must be reflected to predict the deformation of an actual structure exposed to various conditions.

## 1. Introduction

Concrete is a composite composed of cement, aggregate, water, and admixture. It is a nonhomogeneous material owing to its internal pores, which are absent in homogeneous materials such as iron. It may exhibit various deformation characteristics when compared to typical homogeneous materials. It undergoes various deformations based on the presence or absence of an external force. Autogenous shrinkage, drying shrinkage, and shrinkage caused by carbonation may be observed in the absence of an external force. Compressive creep is a physical phenomenon in which the deformation of a member under a continuous load increases over time even without the application of additional loads following the elastic deformation caused by loading; it is typically observed in the presence of an external force [1,2,3,4,5,6].

Recently, the use of high-strength concrete of 80 MPa or higher at construction sites has been increasing owing to the increasing demand for high-rise buildings. In general, high-strength concrete may have different elastic modulus depending on the type of coarse aggregate [7,8]. The elastic modulus is a factor influencing the compressive creep of concrete. The compressive creep of high-strength concrete may vary depending on the type of coarse aggregate [9]. In addition, as the use of high-strength concrete increases, the prediction of the compressive deformation of members using high-strength concrete is gaining significance [10]. The compressive deformation of concrete may vary based on the load condition and concrete mix of the member, and the lack of accurate prediction and construction may produce several problems in concrete structures.

Therefore, various models have been developed to predict the behavior of concrete structures, which are expressed considering the concrete mix and the influence of the environment [11,12,13,14,15]. However, the deformation behavior exhibited by these models varies from that of domestically produced concrete; it has been reported that the difference between the measured and predicted values increases with the increase in age in the case of high-strength concrete [14,15,16].

Moon [14] compared the creep models of the CEB-FIP1990 Model Code, ACI 209R, and Eurocode 2 with the compressive creep test results of 24–60 MPa concrete to analyze the models. Certain differences were observed between the measured and predicted values, and it was reported that it is necessary to construct data that reflect various compressive strengths and loading conditions.

Lee et al. [17] conducted an experimental analysis on the application of models such as KCI 2011, ACI 209R-92, CEB-MC 90–99, B3, and GL2000 to high-strength concrete. They reported that the compressive creep characteristics varied based on the loading age and specimen shape, and that correction was required to apply the existing models to high-strength concrete.

Haranki [18] evaluated the shrinkage and compressive creep of 25–65 MPa concrete and proposed modified formulas by comparing the evaluation results with those of the ACI 209 and CEB-FIP models.

Ojha et al. [19] evaluated the compressive creep of 36–90 MPa concrete and compared the results with those of Bazant’s B-3, ACI, AASHTO, GL2000, and FIB model code 2010.

As explained earlier, further research is required on the applicability of the existing prediction models, and database construction is required for high-strength concrete. Additionally, the shrinkage and compressive creep of high-strength concrete must be predicted through experimental verification and the verification of the construction site conditions. For high-strength concrete, it has been reported that the difference between the measured and predicted values tends to increase with the increase in age [15,20].

Typically, the compressive creep test of concrete requires data for more than six months, and studies that can be used at actual construction sites remain limited [21,22,23].

In this study, the mechanical properties of 70, 80, and 90 MPa high-strength concrete based on limestone and granite coarse aggregates applied to high-rise structures are evaluated, and the shrinkage and compressive creep are analyzed based on the curing conditions. Furthermore, the existing shrinkage and compressive creep models are compared to produce modified formulas.

## 2. Experiment

### 2.1. Experimental Procedure

In this study, high-strength concrete with water/binder (W/B) ratios of 0.24, 0.27, and 0.29 was used. The specimen ID of high-strength concrete was constructed using the compressive strength, curing condition, coarse aggregate condition, and fiber condition (90 MPa). The autogenous shrinkage, drying shrinkage, and compressive creep of high-strength concrete were then evaluated under sealed and unsealed conditions. Table 1 shows the experimental plan.

To control the compressive strength of high-strength concrete, the amount of binder and aggregate was adjusted, and the target slump flow and air contents were satisfied in all specimens. The compressive strength and elastic modulus of high-strength concrete based on limestone and granite coarse aggregates were evaluated to select coarse aggregates for high-strength concrete. Table 2 and Table 3 show the mix proportions of the concrete and the physical–mechanical properties of used material used in this study. Additionally, nylon and polypropylene fibers were mixed at a 1:1 ratio, and 0.1% (*v/v*) was applied to secure the fire resistance performance of high-strength concrete, as shown in Table 4. In this study, the fiber addition and nonaddition conditions were added to 90 MPa concrete to analyze the mechanical and shrinkage characteristics of high-strength concrete based on the presence and absence of fibers.

**Figure 1:**
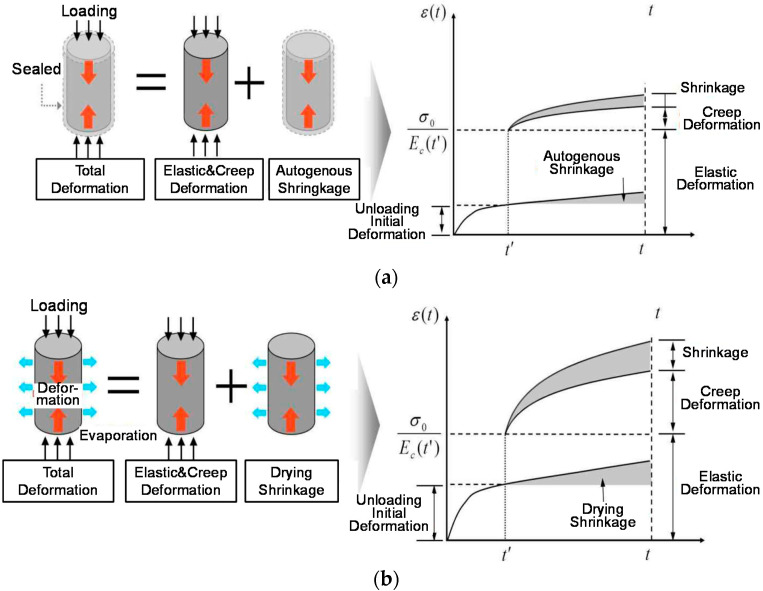
Creep deformation with curing conditions: (**a**) sealed (basic creep); (**b**) unsealed (drying creep).

Figure 1 presents the compressive creep of concrete varies based on the curing conditions of the concrete. Particularly, the drying shrinkage is small, and the autogenous shrinkage in the early ages is greater for high-strength concrete than that for normal-strength concrete. Therefore, the curing conditions and compressive strengths must be considered.

### 2.2. Concrete Specimen Production and Shrinkage Characteristics Evaluation Method

**Figure 2:**
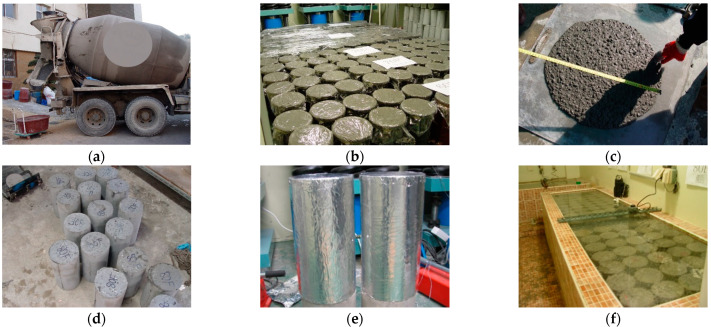
Specimen production and test preparation: (**a**) concrete mixing in batcher plant; (**b**) evaluation of fresh concrete; (**c**) production of specimens; (**d**) demolding of the specimen; (**e**) curing; (**f**) pretreatment of specimen.

**Figure 3:**
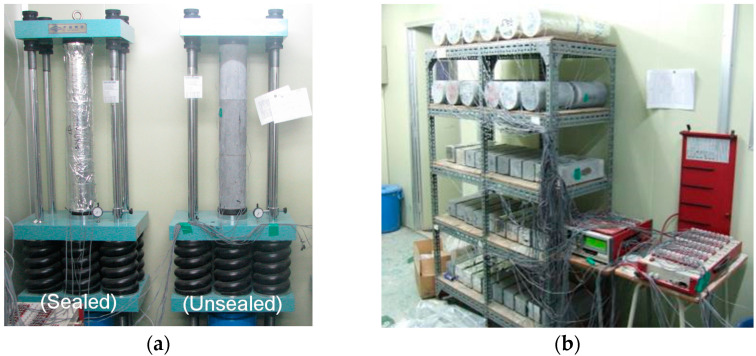
Test overview of high-strength concrete: (**a**) creep test; (**b**) shrinkage test.

Figure 2 illustrates the concrete specimen production process. The high-strength concrete used in this study was applied to high-rise buildings, and specimens were fabricated using the concrete produced in the batcher plant for testing under field conditions. The compressive strength and elastic modulus were evaluated in accordance with the “KS F 2405 Standard test method for compressive strength of concrete” and “KS F 2438 Standard test method for static modulus of elasticity and Poisson’s ratio in compression of cylindrical concrete specimens”. Additionally, autogenous shrinkage and drying shrinkage were evaluated in accordance with the “KS F 2586 Standard test for autogenous shrinkage and expansion of cement paste, mortar and concrete” and “KS F 2424 Standard test method for length change of mortar and concrete”. The specimens required for the compressive creep test were fabricated with a size of Ø150 × 300 mm in accordance with the “KS F 2453 Standard test method for creep of concrete in compression”. Dry curing was performed after concrete pouring at a temperature of 20 ± 2 °C for 24 h. The unsealed specimens were then demolded and subjected to wet curing at the same temperature for seven days. They were subjected to dry curing at a relative humidity of 50 ± 10% for 28 days. Additionally, the sealed specimens were cured by sealing them with plastic wrap and aluminum tape after demolding. In the compressive strength and modulus test, plastic wrap and aluminum tape were removed before proceeding. Figure 3 illustrates the process of measuring autogenous shrinkage, drying shrinkage, and compressive creep.

**Figure 4:**
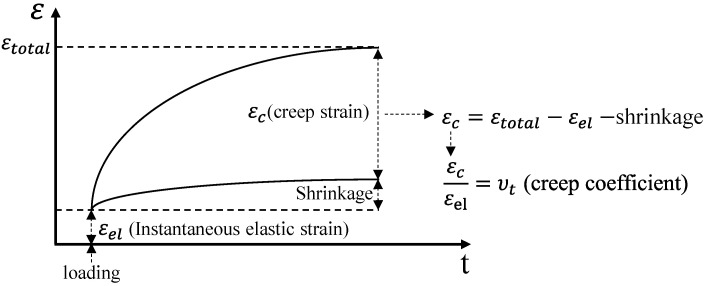
Concept of creep strain under load and creep coefficient.

In the compressive creep test, the loading level was set to 40% of the compressive strength at 28 days of age, and the measurements were performed for two years after loading. The creep strain was calculated by subtracting the instantaneous elastic strain and shrinkage immediately after loading from the total strain measured in the compressive creep test, as shown in Figure 4. In this study, the compressive creep coefficient was also calculated by dividing the creep strain by the instantaneous elastic strain, as shown in Equation (1) [11]. It was calculated to compare the compressive creep of concrete with different compressive strengths under different loads.

### 2.3. Shrinkage and Creep Models

The models used to predict the shrinkage and compressive creep of concrete have been proposed and modified by various researchers in previous studies. In this study, the prediction models that are mainly examined in South Korea were selected and compared with the experimental results for high-strength concrete.

The autogenous shrinkage and drying shrinkage of high-strength concrete were compared with the CEB code [12] and Eurocode 2 [13]. ACI 209 [8] was selected for the compressive creep. The compressive creep coefficient presented in ACI 209 is the product of the ultimate creep coefficient determined by the age and environmental factors at the time of loading and the formula that expresses the occurrence of compressive creep over time after loading, as shown in Equations (2) and (3). The applicable correction factors for the ultimate creep coefficient are composed of functions that add to or subtract from the default value of “1”. The ultimate creep coefficient does not exceed this value since the factors are multiplied by a constant of 2.35.

(1) $$\upsilon_{t}=\frac{\varepsilon_{c}}{\varepsilon_{\mathrm{el}}}$$

where

$\upsilon_{t}$: creep coefficient;
$\varepsilon_{c}$: creep strain;
$\varepsilon_{\mathrm{el}}$: instantaneous elastic strain.

(2) $$\upsilon_{t}=\frac{t^{0.6}}{10+t^{0.6}}\upsilon_{u}$$

(3) $$\upsilon_{u}=2.35\times \gamma_{c}$$

where

$\upsilon_{t}$: creep coefficient;
t: days after loading;
$\upsilon_{u}$: ultimate creep coefficient;
$\gamma_{c}$: applicable correction factors.

## 3. Experimental Results and Discussion

### 3.1. Compressive Strength and Elastic Modulus

**Figure 5:**
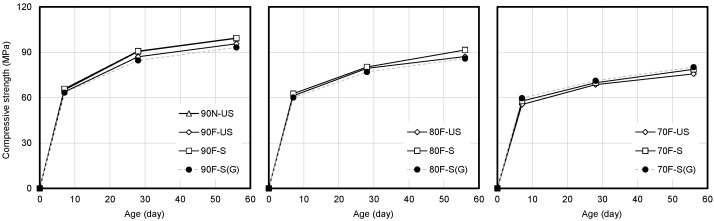
Compressive strength of high-strength concrete based on limestone and granite.

**Figure 6:**
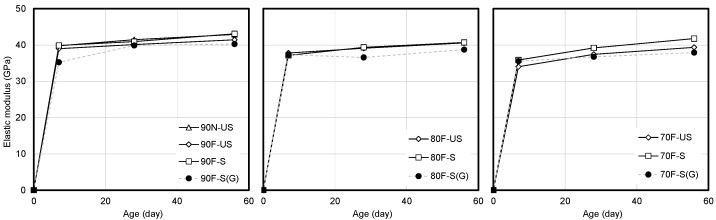
Elastic modulus of high-strength concrete based on limestone and granite.

Figure 5 shows the compressive strength of high-strength concrete. When the compressive strengths of 90, 80, and 70 MPa high-strength concrete based on limestone coarse aggregate at 28 days of age are measured, 87.0, 79.4, and 87.0 MPa are obtained under the unsealed condition, and 90.8, 80.3, and 69.9 MPa are obtained under the sealed condition, indicating that the sealed condition produces higher compressive strength. When the granite coarse aggregate is used, 84.6, 77.00, and 71.20 MPa are measured under the sealed conditions. Figure 6 shows the elastic modulus of high-strength concrete based on limestone coarse aggregate for which, the elastic modulus is measured to be approximately 1–4% higher under the sealed condition than under the unsealed condition at 28 days of age. The elastic modulus is mostly observed at 10 days of age and shows a tendency to converge.

**Figure 7:**
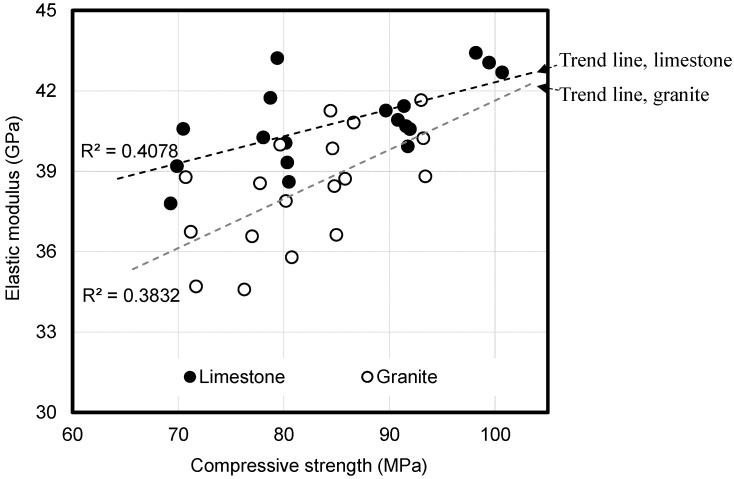
Comparison of compressive strength and elastic modulus of high-strength concrete at 28 and 56 days of age.

Figure 7 presents the comparison of the compressive strength and elastic modulus of high-strength concrete at 28 and 56 days of age. The material value trend line of high-strength concrete based on limestone coarse aggregate is observed to be higher. This tendency is produced by a phenomenon that can be observed in high-strength concrete based on limestone coarse aggregate. This phenomenon is reportedly caused by the mineral properties of limestone and the physicochemical properties of the aggregate [24,25,26]. The limestone coarse aggregate is used in this study because the elastic modulus significantly affects the deformation characteristics of concrete.

When a comparison is performed for 90 F to compare the compressive strength based on the fiber addition, it is observed that the compressive strength tends to decrease by approximately 3% when fibers are added [27]. Additionally, the compressive strength and elastic modulus are observed to be higher under the sealed condition than under the unsealed condition. It is observed that the cement hydration reaction can continuously occur under the sealed condition when compared to the unsealed condition because the moisture in concrete cannot be discharged [28,29].

### 3.2. Autogenous and Drying Shrinkage

In this study, the shrinkage behavior was evaluated until 180 days of age to compare and examine shrinkage behavior at early ages.

**Figure 8:**
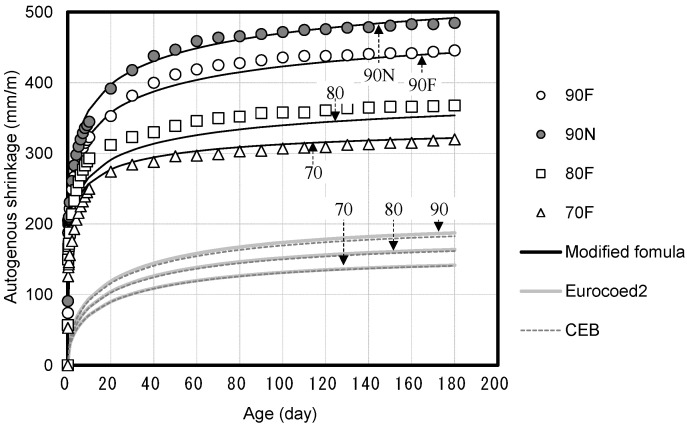
Autogenous shrinkage of high-strength concrete and code comparison.

Figure 8 presents the autogenous shrinkage of the high-strength concrete based on the compressive strength. The autogenous shrinkage increases with the increase in the compressive strength of the concrete. Particularly, approximately 70% of the total autogenous shrinkage is observed until seven days of age. The effect of the sealed condition (basic creep) on the compressive creep in which the autogenous shrinkage is considered is determined to be insignificant because most of the autogenous shrinkage develops at an early age before loading. As high-strength concrete has a low W/B unlike normal-strength concrete, self-desiccation is observed, which is a phenomenon where the internal moisture is lost due to the continuous hydration of unhydrated cement [30]. This causes a large autogenous shrinkage of high-strength concrete compared with normal-strength concrete, and it is important for deformation prediction.

(4) $$\epsilon_{as}\left(t\right)=f\times \gamma \times \epsilon_{\infty }\left(W/B\right)\times \beta \left(t\right)$$

(5) $$\epsilon_{\infty }\left(W/B\right)=3070\;\exp\left[-7.2\left(W/B\right)\right]$$

(6) $$\beta \left(t\right)=1-\exp\left[-a{\left(t-t_{0}\right)}^{b}\right]$$

(7) a=5.2843×(W/B)−0.7576

(8) b=−1.3037×(W/B)−0.9937

where

$\epsilon_{as}\left(t\right)$: autogenous shrinkage with age (×10^−6^);
$\epsilon_{\infty }\left(W/B\right)$: maximum amount of autogenous shrinkage with W/B (×10^−6^);
f: fiber mixing constant (range of this study: 0.9 when mixed, 1.0 without mixing);
γ: aggregate constant (granite 1.0, limestone 1.087);
β(t): function that represents the progress of autogenous shrinkage;
a,b: experimental constants (Table 5);
t: age (days);
$t_{0}$: initial set time (days).

The results of the autogenous shrinkage measurement of high-strength concrete were compared with the autogenous shrinkage prediction formulas of Eurocode 2 and the CEB code. The autogenous shrinkage measurements of high-strength concrete were approximately twice as high as the autogenous shrinkage values predicted using Eurocode 2 and the CEB code.

Miyazawa and Tazawa [31] proposed an autogenous shrinkage prediction model that reflects the characteristics of high-strength concrete in which most of the autogenous shrinkage is observed at the early ages. In this study, Equations (4)–(8) and Table 5. are proposed using the model proposed by Miyazawa and Tazawa by modifying constants to fit the measured autogenous shrinkage values of high-strength concrete.

**Figure 9:**
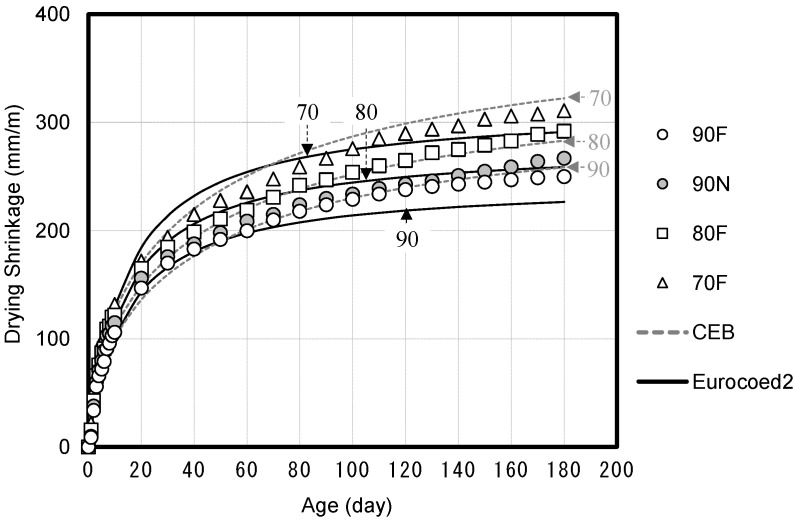
Drying shrinkage of high-strength concrete and code comparison.

Figure 9 shows the drying shrinkage of high-strength concrete based on the compressive strength. The drying shrinkage decreases with the increase in the compressive strength of concrete, and approximately 70% of the total shrinkage is observed between 90 and 100 days of age. The drying shrinkage is observed to decrease with the increase in the compressive strength of concrete due to the presence of a small amount of free water in the concrete, as W/B is low and the unit water content is small.

Therefore, the compressive creep under the unsealed condition (drying creep) is significantly affected by the drying shrinkage, and this influence is expected to decrease as the compressive strength increases. Additionally, it is observed that the measured drying shrinkage values of high-strength concrete are similar to the predicted drying shrinkage values of Eurocode 2 and the CEB code.

When fibers are added, the autogenous shrinkage and drying shrinkage are reduced by approximately 2–4%. This is because the fibers restrict the concrete matrix and inhibit moisture movement [10].

### 3.3. Compressive Creep Coefficient

**Figure 10:**
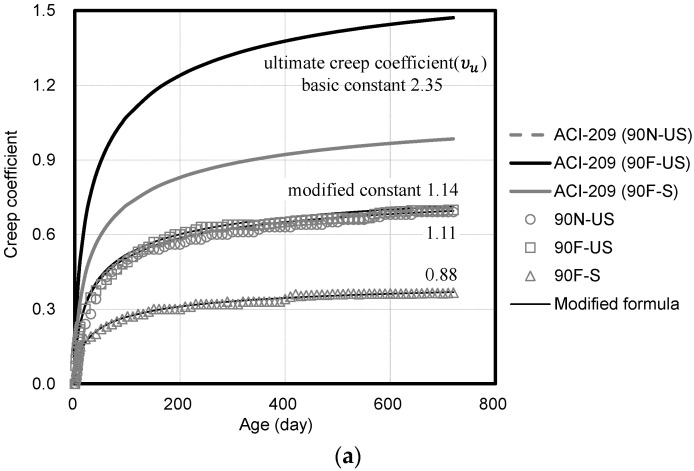

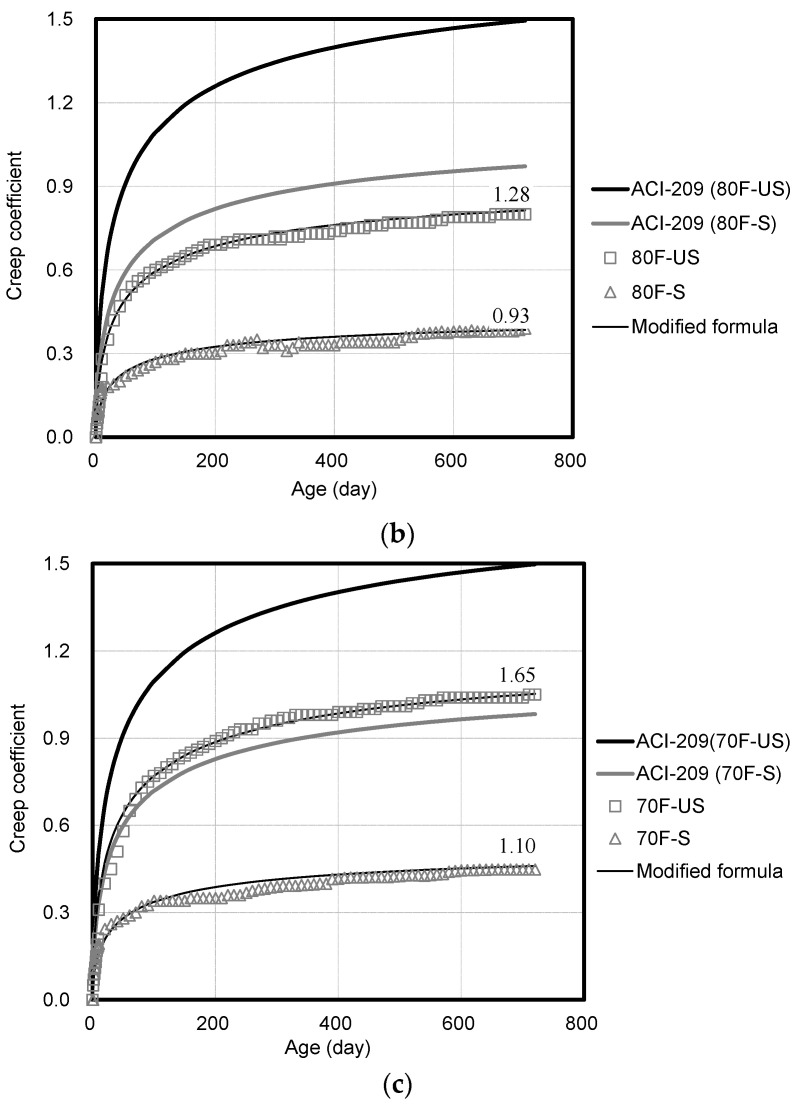
Compressive creep coefficient of high-strength concrete: (**a**) 90 MPa; (**b**) 80 MPa; (**c**) 70 MPa.

Figure 10 shows the compressive creep coefficient of high-strength concrete based on the compressive strength and curing conditions. For the compressive creep coefficient, the measured values are compared with the predicted values of ACI 209.

At 720 days of age, the compressive creep coefficients of (90 N), 90 F, 80 F, and 70 F are 0.45, 0.39, and 0.36 under sealed conditions and (0.69), 0.71, 0.81, and 1.05, respectively, under unsealed conditions. The compressive creep coefficient under the unsealed condition is approximately 1.58 to 2.91 times higher than that under the sealed condition. This can be attributed to the fact that no drying shrinkage is observed under the sealed conditions. The difference in the compressive creep coefficient decreases with the increase in the compressive strength. This is because the drying shrinkage decreases with the increase in the compressive strength. Loading is applied to concrete after 28 days of age during the laboratory tests. However, it is necessary to consider both autogenous shrinkage and drying shrinkage for actual buildings because concrete structures are fabricated based on the construction period.

Under the unsealed condition, the compressive creep coefficient of 90 MPa concrete increases by approximately 3% owing to fiber addition. This can be attributed to the fact that the interfacial structure of concrete is weakened and the creep strain increases owing to the addition of fibers with a lower elastic modulus than that of concrete [32,33].

**Figure 11:**
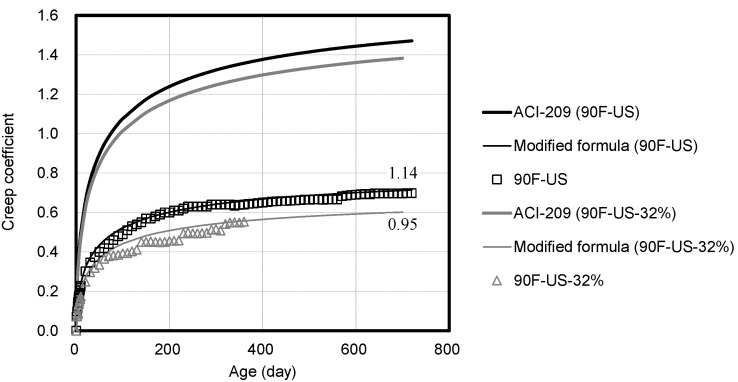
Comparison of compressive creep coefficients based on 40% and 32% load.

**Figure 12:**
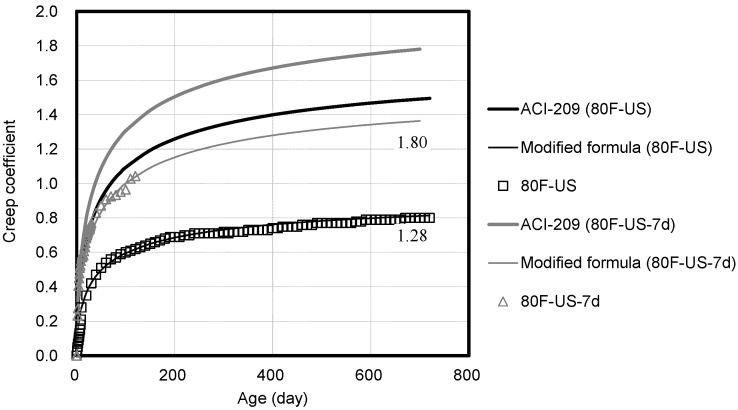
Comparison of compressive creep coefficients based on the age of loading of 28 days and 7 days.

Figure 11 shows the compressive creep coefficient corresponding to the loading level. The creep strain at the beginning of the loading and the overall creep strain decreases with the decrease in the loading level. The loading level must be considered because the load applied to the concrete structural members may vary depending on the building. Figure 12 shows the compressive creep coefficient corresponding to the loading age. The compressive strength of high-strength concrete is measured at seven days of age, and 40% of it is applied to provide the same conditions. At the loading age of seven days, the initial creep strain is observed to be larger than the loading at 28 days. Additionally, the value predicted by using ACI 209 varies because ACI 209 considers the age of loading. However, the value predicted by using ACI 209 varies from the measured compressive creep coefficient values of high-strength concrete.

When the compressive creep coefficient of high-strength concrete is analyzed using ACI 209, the predicted values are evaluated to be approximately 1.42 to 2.07 times higher than the measured values under the unsealed condition and approximately 2.13 to 2.65 times higher under the sealed condition. The prediction of compressive creep using ACI 209 is the method mainly used in Korea. However, this method cannot reflect the compressive creep characteristics of high-strength concrete with a strength of 70 MPa or higher. Additionally, the increase in the difference in the compressive creep coefficient is attributed to the high elastic modulus of high-strength concrete based on limestone coarse aggregate [34].

**Figure 13:**
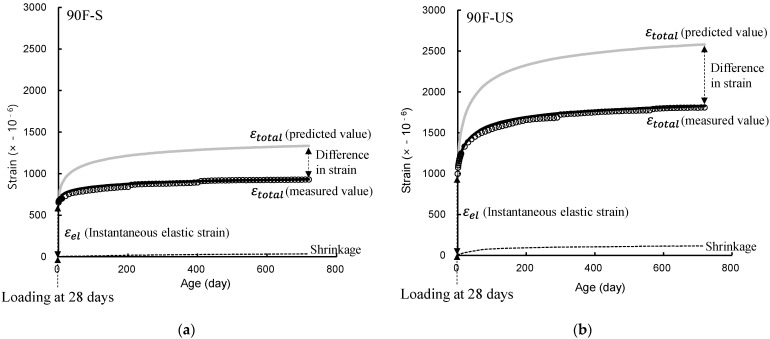
Total strain of 90 MPa concrete calculated from compressive creep coefficients that corrected through measured values and predicted through ACI 209: (**a**) sealed; (**b**) unsealed.

Additionally, the total strain calculated by the compression creep coefficient predicted by using ACI 209 is significantly different from the measured total strain regardless of the curing conditions, as shown in Figure 13. The total strain of high-strength concrete predicted by using ACI 209 may differ from the compressive strain behavior of the structures. Therefore, this study proposes constants that can modify the constant 2.35 of the ultimate creep coefficient of the ACI 209 model. The constants of the ultimate creep coefficient are derived by reflecting the measured compressive creep values of high-strength concrete in consideration of the curing condition and compressive strength and are shown in Equation (9) and Table 6.

(9) $$\upsilon_{u}\prime =a\times \gamma_{c}$$

where

$\upsilon_{u}\prime$: modified ultimate creep coefficient;
a: modified constants;

## 4. Conclusions

1. The compressive strength and elastic modulus of high-strength concrete were evaluated to be approximately 2% higher under the sealed condition than under the unsealed condition based on the curing conditions. This is attributed to the moisture conserved in the concrete under sealed conditions. Additionally, the compressive strength and elastic modulus of the high-strength concrete increased by approximately 4% when limestone coarse aggregate was used.
2. The autogenous shrinkage increases, and the drying shrinkage decreases with the increase in the compressive strength of the concrete. Particularly, the autogenous shrinkage of high-strength concrete increases owing to the self-desiccation caused by the hydration of unhydrated cement, which cannot be implemented by the existing models. Autogenous shrinkage and drying shrinkage occur simultaneously under the unsealed conditions in actual structures. Therefore, both autogenous shrinkage and drying shrinkage must be considered to predict the overall shrinkage of high-strength concrete. Furthermore, modified formulas were derived using the autogenous shrinkage prediction model proposed by a previous study, which reflected the autogenous shrinkage development characteristics of high-strength concrete.
3. The compressive creep coefficient of high-strength concrete was evaluated to be approximately 1.58 to 2.91 times higher under the unsealed condition than under the sealed condition based on the curing conditions and was also affected by the compressive strength, loading level, and loading age. Additionally, the coarse aggregates affect the elastic modulus and compressive creep of high-strength concrete, which varies depending on various conditions, which cannot be reflected by the existing models. The shrinkage and compressive creep characteristics must be reflected to improve the prediction accuracy for the deformation of high-strength concrete because actual structures are exposed to more conditions than in laboratory tests. Therefore, ACI-209 proposed modified formulas to derive the ultimate creep coefficient by reflecting the compressive creep characteristics of high-strength concrete based on the curing condition and compressive strength.
4. High-strength concrete has different internal structures because it contains more binders than normal-strength concrete. Therefore, the shrinkage and compressive creep of the main structural members must be accurately predicted to ensure structural stability in high-rise buildings where high-strength concrete is used. The accurate prediction of the deformation characteristics of high-strength concrete requires the construction of databases considering conditions such as compressive strength, curing condition, and coarse aggregate. In addition, future studies should investigate the correspondence between the compressive deformation of concrete members of buildings and the predicted values of the compressive deformation of concrete based on material values.

## Figures and Tables

**Table 1:** Experimental plan.

W/B	Specimen ID ^1^	Curing Condition	Test Item
0.24	90N-US 90F-US 90F-S 90F-S(G) ^4^ 90F-US-32% ^2^	Sealed Unsealed	Compressive strength (MPa) Elastic modulus (MPa) Autogenous shrinkage Drying shrinkage Compressive creep
0.27	80F-US 80F-S 80F-S(G) ^4^ 80F-US-7d ^3^		
0.29	70F-US 70F-S 70F-S(G) ^4^		

^1^ F: Fiber addition condition, N: Fiber nonaddition condition, S: Sealed condition, US: Unsealed condition. ^2^ 32%: Load (default: 40% of the compressive strength). ^3^ 7d: Loading age (default: 28 days). ^4^ For comparison with limestone, only compressive strength and elastic modulus are evaluated.

**Table 2:** Mix proportion.

f_ck_	W/B	Slump Flow (mm)	Air (%)	S/a (%)	Unit Weight ^1^ (kg/m^3^)						
					W	C	FA	SF	S	LG	GG
90	0.24	650 ± 20	1.6	47.5	155	481	130	39	745	835	829
80	0.27	650 ± 20	1.7	48.0	158	443	114	23	770	865	856
70	0.29	650 ± 20	1.8	49.0	163	418	110	22	815	875	868

^1^ W: Water, C: Cement, FA: Fly ash, SF: Silica fume, S: Fine aggregate, LG: Limestone, GG: Granite.

**Table 3:** Physical–mechanical properties of used material.

Material	Physical Properties
Cement	Ordinary Portland cement (OPC, density: 3.15 g/cm^3^, specific surface area: 3440 cm^2^/g)
Fly ash	Density: 2.30 g/cm^3^, specific surface area: 3228 cm^2^/g
Silica fume	Density: 2.22 g/cm^3^, specific surface area: 200,000 cm^2^/g
Fine aggregate	Sea sand (size: 5 mm, density: 2.56 g/cm^3^, absorption: 1.01%)
Coarse aggregate	Crushed limestone (size: 20 mm, density: 2.69 g/cm^3^, absorption: 0.32%, solid volume percentage: 60.80%) Crushed granite (size: 20 mm, density: 2.67 g/cm^3^, absorption: 0.35%, solid volume percentage: 55.42%)
Superplasticizer	Polycarboxylic-based superplasticizer (specific gravity: 1.05 ± 0.05, pH: 5.0 ± 1.5)

**Table 4:** Mechanical properties of fibers.

Fiber Type	Length (mm)	Diameter (mm)	Aspect Ratio	Density (g/cm^3^)	Tensile Strength (MPa)	Extension (%)	Elastic Modulus (GPa)	Melting Point (°C)
Nylon	12.49	23.8	524.8	1.14	903.2	18.9	5.0	219.9
Polypropylene	19.17	37.0	518.1	0.91	611.4	21.7	5.8	167.7

**Table 5:** Experimental constants a, b in Equation (7,8).

f_ck_	Constant	
	a	b
90	0.6	
80	0.65	0.25
70	0.8	

**Table 6:** Modified constants a in Equation (9).

Condition	Basic	90N	90F	90F (32%)	80F	80F (7d)	70F
Sealed	2.35	-	0.88	-	0.93	-	1.10
Unsealed		1.11	1.14	0.95	1.28	1.80	1.65
